# Hospital discharge data is not accurate enough to monitor the incidence of postpartum hemorrhage

**DOI:** 10.1371/journal.pone.0246119

**Published:** 2021-02-03

**Authors:** Diana Walther, Patricia Halfon, Romain Tanzer, Bernard Burnand, Moira Robertson, Yvan Vial, David Desseauve, Marie-Annick Le Pogam

**Affiliations:** 1 Department of Epidemiology and Health Systems, Center for Primary Care and Public Health (Unisanté), University of Lausanne, Lausanne, Switzerland; 2 Data Science and Research Unit, Lausanne University Hospital (CHUV), Lausanne, Switzerland; 3 Department of Anesthesiology, Lausanne University Hospital (CHUV), Lausanne, Switzerland; 4 Department Woman-Mother-Child, Lausanne University Hospital (CHUV), Lausanne, Switzerland; Karolinska Institutet, SWEDEN

## Abstract

**Introduction:**

Postpartum hemorrhage remains a leading cause of maternal morbidity and mortality worldwide. Therefore, cumulative incidence of postpartum hemorrhage and severe postpartum hemorrhage are commonly monitored within and compared across maternity hospitals or countries for obstetrical safety improvement. These indicators are usually based on hospital discharge data though their accuracy is seldom assessed. We aimed to measure postpartum hemorrhage and severe postpartum hemorrhage using electronic health records and hospital discharge data separately and compare the detection accuracy of these methods to manual chart review, and to examine the temporal trends in cumulative incidence of these potentially avoidable adverse outcomes.

**Materials and methods:**

We analyzed routinely collected data of 7904 singleton deliveries from a large Swiss university hospital for a three year period (2014–2016). We identified postpartum hemorrhage and severe postpartum hemorrhage in electronic health records by text mining discharge letters and operative reports and calculating drop in hemoglobin from laboratory tests. Diagnostic and procedure codes were used to identify cases in hospital discharge data. A sample of 334 charts was reviewed manually to provide a reference-standard and evaluate the accuracy of the other detection methods.

**Results:**

Sensitivities of detection algorithms based on electronic health records and hospital discharge data were 95.2% (95% CI: 92.6% 97.8%) and 38.2% (33.3% to 43.0%), respectively for postpartum hemorrhage, and 87.5% (85.2% to 89.8%) and 36.2% (26.3% to 46.1%) for severe postpartum hemorrhage. Postpartum hemorrhage cumulative incidence based on electronic health records decreased from 15.6% (13.1% to 18.2%) to 8.5% (6.7% to 10.5%) from the beginning of 2014 to the end of 2016, with an average of 12.5% (11.8% to 13.3%). The cumulative incidence of severe postpartum hemorrhage remained at approximately 4% (3.5% to 4.4%). Hospital discharge data-based algorithms provided significantly underestimated incidences.

**Conclusions:**

Hospital discharge data is not accurate enough to assess the incidence of postpartum hemorrhage at hospital or national level. Instead, automated algorithms based on structured and textual data from electronic health records should be considered, as they provide accurate and timely estimates for monitoring and improvement in obstetrical safety. Furthermore, they have the potential to better code for postpartum hemorrhage thus improving hospital reimbursement.

## Introduction

Postpartum hemorrhage (PPH) remains the leading cause of severe maternal morbidity and avoidable mortality, accounting for 27% of maternal deaths worldwide [1]. PPH results in frequent and note-worthy physical and psychological consequences [2–5]. Its association with longer hospital stays and readmissions creates a non-negligible economic burden [6, 7]. The global incidence of PPH and severe PPH, estimated at 6% and 2% respectively, varies greatly by setting [8] due to differences in maternal- and pregnancy-related characteristics and quality of care [9–12]. As suggested by recent quality improvement initiatives, PPH monitoring is the most relevant means to better understand and devise strategies to mitigate this obstetrical complication and then to evaluate the impact of obstetrical safety improvement interventions [13].

Hospital discharge data (HDD) is still widely used in the monitoring of and comparisons in PPH incidence nationally and internationally [14–20]. Yet, determining the degree to which variation in outcome indicators such as PPH incidence reflects obstetrical safety remains a challenge due to differences in data quality, PPH definitions, methods of measuring blood loss, case-mix (i.e. distribution of risk factors among parturients) and chance [21]. In Switzerland HDD is required for hospital billing and generated by professional coders who attribute diagnostic and procedure codes based on medical records according to national coding rules and classifications. However, limited accuracy in code attribution may alter case detection and bias both measures of incidence and comparisons [21, 22]. Definitions of PPH also vary widely; they are typically based on estimated blood loss (EBL), drop in hemoglobin (ΔHb) or clinical judgment concerning tolerance of blood loss. Quantification of blood loss is challenging despite the wide range of techniques available [23]. In addition, the EBL thresholds used to categorize blood loss in PPH vary according to the route of delivery. For example, the PPH thresholds used for vaginal and caesarean delivery are most commonly 500 ml and 1000 ml, respectively. These thresholds also vary for severe PPH [11, 24]. Finally, Hb is not always routinely measured, acute bleeding does not lead to an immediate drop in Hb, and change in Hb is influenced by multiple factors including transfusion, hemodilution and a variety of pathologies. This situation is further complicated by the lack of consensus regarding what differentiates PPH from severe PPH [11, 24].

Changes in PPH definitions are ongoing [3, 25] and there has only been limited efforts to assess the accuracy of HDD-based PPH indicators [26–28]. Within this challenge-ridden landscape, the increasing availability of electronic health records (EHRs) may present an opportunity in PPH surveillance. The aim of this study was to detect PPH/severe PPH using EHR on one hand and using HDD on the other hand, and to estimate the accuracy (sensitivity and specificity) of each detection method (EHR-based detection and HDD-based detection) using manual chart review as the "gold standard." A secondary aim was to examine variations over time in cumulative incidence estimated over four-month periods.

## Materials and methods

### Population and data

We retrospectively included all parturients having had a singleton delivery between 2014 and 2016 in one of the largest Swiss University Hospitals. Parturients included were those not transferred to another hospital during or after delivery, and who had signed a general consent for secondary use of their medical data for research (n = 8114). For each delivery included, HDD consisted of date of delivery, diagnoses coded with the International Classification of Diseases, 10th Revision, German Modification (ICD-10-GM), procedures coded with the Swiss classification for surgical procedures (CHOP), number of hours spent in an intensive care unit, and in-hospital death. EHR contained both structured and unstructured data; it was used in full for manual chart review and in part in the EHR-based case detection as described later in the methods section. Structured EHR data consisted of patient characteristics, categorical delivery room EBL (cumulative EBL in the delivery room only; <500, 500–1000, 1000–1500, >1500 ml), transfusion data and Hb before, during and after delivery. All clinical data was collected as part of daily routine care by midwifes, nurses and physicians. The medication prescribed and administered during the stay were not available. Unstructured EHR data included discharge letters and operative reports. Following hospital guidelines, EBL was quantified in the delivery room using graduated under buttocks fluid collection pouches and fluid aspiration systems for vaginal births and caesareans respectively and by weighing blood soaked material and clots at delivery and on the ward. We excluded women with missing categorical delivery room EBL (n = 210), which led to an analytical sample of 7904 singleton deliveries (see S1 and S2 Tables).

### Definitions

Based on the literature, definitions of PPH applying to this analysis feature in Table 1. We considered any occurrence of PPH until hospital discharge, including early (within 24 hours postpartum) and late PPH (occurring after 24 hours).

**Table 1:** Definitions of PPH and severe PPH, by type of delivery.

	PPH vaginal births	PPH caesareans	Severe PPH vaginal births	Severe PPH caesareans	
Local hospital definition	EBL ≥ 500 ml or clinical diagnosis*	EBL ≥ 1000 ml or clinical diagnosis*	EBL ≥ 1000 ml or clinical diagnosis*	EBL ≥ 1500 ml or clinical diagnosis*	
Criteria guiding chart review	EBL ≥ 500 ml, ΔHb >2 g/dl, clinical diagnosis* or drug treatment for PPH^†^	EBL ≥ 1000 ml, ΔHb >2 g/dl, clinical diagnosis* or drug treatment for PPH^†^	EBL ≥ 1000 ml, ΔHb ≥ 4 g/dl, clinical diagnosis* or interventional treatment or transfusion of ≥ 4 PRBCs^†^	EBL ≥ 1500 ml, ΔHb ≥ 4 g/dl, clinical diagnosis* or interventional treatment or transfusion of ≥ 4 PRBCs^†^	
EHR definition	EBL ≥ 500 ml	EBL ≥ 1000 ml	EBL ≥ 1000 ml or EBL ≥ 500 ml & ΔHb ≥ 4g/dl	EBL ≥ 1500 ml or EBL ≥ 1000 ml & ΔHb ≥ 4g/dl	
HDD definition	ICD-10-GM codes	ICD-10-GM codes	ICD-10-GMor CHOP codes or intensive care or death		ICD-10-GM or CHOP codes or intensive care or death

CHOP: Swiss classification for surgical procedures; EBL: estimated blood loss; EHR: electronic health record; ΔHb: peripartum change in hemoglobin = (last antenatal Hb within 30 days if available, otherwise maximum Hb on delivery day)–(nadir post-delivery Hb during hospitalization); HDD: hospital discharge data; ICD-10-GM: International Classification of Diseases, 10^th^ Revision, German Modification; PPH: postpartum hemorrhage; PRBCs: packed red blood cells (one unit = 500 ml, hematocrit 0.6 ± 0.1)

*Clinical tolerance and rapidity of blood loss can result in a clinical diagnosis of PPH/severe PPH before the indicated cut-offs are reached.

^†^Hospital PPH guidelines recommended specific pharmacological/surgical interventions and transfusions based on the severity of bleeding. We therefore used treatment type as a guide to identify PPH/severe PPH during chart review.

### Reference standard, chart review

In order to represent a wide range of PPH risks, and to limit the sample size for retrospective chart review [29], we stratified vaginal and cesareans births separately, each into 16 levels of PPH risk, based on structured data from the EHR or HDD (see *S1 and S2 Tables*). The levels of stratification were based on the presence or absence of the following criteria: 1) categorical EBL measured in the delivery room (≥ 500 ml versus < 500 ml for vaginal births and ≥ 1000 ml versus < 1000 ml for caesarians); 2) change in peripartum Hb (> 2 g/dl versus ≤ 2g/dl or not known); 3) the presence of at least one of the following factors related to PPH (morbidly adherent placenta, uterine exploration, manual removal of retained placenta, or repair of a genital tract injury) and; 4) the presence of at least one of the following criteria for severity (categorical delivery room EBL >1000 ml in vaginal births or >1500 ml in caesareans, change in peripartum Hb ≥4 g/dl, transfusion of ≥4 units of packed red blood cells (one unit = 500 m, hematocrit 0.6 ± 0.1), arterial embolization, ligature or occlusion, hysterectomy or uterine tamponade). Change in peripartum Hb was calculated as: (last antenatal Hb within 30 days if available, otherwise maximum Hb on delivery day)–(nadir post-delivery Hb during hospitalization). In strata with less than 10 births, all were included in the manual chart reviewed. In strata with 10 or more births, at least 10 charts per strata were randomly sampled for review. A trained physician performed an in-detail review of the 334 sampled charts, using the full EHR, which included medical, operative, and anesthesia letters, reports and notes, drug prescriptions and partograms. Unclear cases were extensively peer reviewed to obtain consensus regarding the presence or absence of PPH and severe PPH. Two cases were excluded due to inconclusive data, leaving 332 charts for further analysis. Results from chart review were considered to be the reference standard.

### EHR-based case detection

Numerical values for EBL or descriptions of normal EBL, when available, were extracted via text mining from all discharge letters and available operative reports. Text mining is the process of exploring and analyzing large amounts of unstructured text data aided by software that can identify concepts, patterns, topics, keywords and other attributes in the data. When EBL was present as a range, we kept the mean of the upper and lower limits (i.e. 200–300 ml became 250 ml). When more than one EBL featured, we kept the highest since cumulative blood loss was of interest. When no numerical value was present, we considered EBL to be normal if it was described as such. Text mining was performed using Python programming language version 3.6.3. (Python Software Foundation, www.python.org). When text mining was unable to extract the data described above, the categorical delivery room EBL from the structured EHR data was used. Change in peripartum Hb was calculated in the same way as described above: (last antenatal Hb within 30 days if available, otherwise maximum Hb on delivery day)–(nadir post-delivery Hb during hospitalization).

### HDD-based case detection

PPH was identified using ICD-10 GM diagnostic codes for postpartum hemorrhage (O70), more specifically PPH due to retained/adherent placenta (O72.0), uterine atony (O72.1), late PPH (O72.2) and coagulation defects (O72.3). Among those with PPH, severe PPH was identified using Swiss classification for surgical procedures (CHOP) codes for infusion of vasopressive agents or transfusion of blood products, arterial ligation, occlusion or embolization, uterine tamponade or, hysterectomy, or using diagnostic codes for hemorrhagic shock or discharge (death) or transfer data (to intensive care after delivery). *Details feature in the S3 Table*.

### Statistical analysis

In descriptive bivariate analysis, using Pearson’s chi-squared test, we assessed differences in the proportions of PPH and severe PPH cases by year, delivery type, maternal age, gestational age, gravidity, parity, and birthweight in turn. To estimate EHR-based and HDD-based case detection accuracy in the population from the stratified design we first estimated sensitivity and specificity in each stratum *i (*n = 32*)*. Estimates for the population were a weighted average of parameters, where the weights (W_i_) were the population proportion of all cases (PPH, severe PPH) belonging to stratum *i* for sensitivity and the proportion of all non-cases (parturient without PPH, without non severe PPH) belonging to stratum *i* for specificity [29, 30]. Asymptotic 95% confidence intervals around sensitivity and specificity estimates were constructed under a normal distribution where variance of the stratification design was ∑W _i_^2^ Var(Se_i_ or Sp_i_) (*i* = 1 to 32). Cohen’s Kappa coefficient and its asymptotic 95% confidence interval assessed concordance between HDD and EHR-based case detection. To study trends in PPH cumulative incidence and the proper allocation of PPH codes over time, we first calculated PPH and severe PPH cumulative incidence over four-month periods using EHRs and HDD and plotted them over time. We then performed one multivariable logistic regression with EHR-based PPH and one with severe PPH as outcomes and included quadrimester of delivery by year as an explicative variable controlling for maternal age, gravidity, parity, vaginal/cesarean delivery and birth weight. Using the EHR-based case detection as a reference standard, we determined if HDD detected PPH and severe PPH correctly for each delivery. Using these results, we then performed one multivariable logistic regression with *correct allocation of PPH* and one with *correct allocation of severe PPH* as outcomes and included quadrimester of delivery by year as an explicative variable. We then performed a sensitivity analysis, controlling for maternal age, gravidity, parity, vaginal/cesarean delivery and birth weight and compared results. Statistical analyses were conducted using STATA 15 (StataCorp. 2015. Stata: Release 15. Statistical Software. College Station, TX: StataCorp LP.)

### Ethical approval

This study was approved by the Vaud cantonal ethics committee (CER VD authorization 2016–02120, December 27, 2016).

## Results

### EHR- and HDD-based PPH/severe PPH and population characteristics

Characteristics of the study population are described in Table 2. Cumulative incidence of PPH and severe PPH were about twice as high based on the EHR compared to HDD; that is, 12.5% (CI; 11.8 to 13.3) vs 6.7% (CI; 6.2 to 7.3) and 3.9% (CI; 3.5 to 4.4) vs 1.6% (CI; 1.4 to 1.9). In bivariate analyses, using the EHR-based case detection, PPH was more frequent in earlier years, in vaginal and pre- and post-term deliveries, and in presence of a birthweight >4500g. Severe EHR-based PPH was more frequent in women over 35 years old, pre- and post-term deliveries and multigravida (≥4 pregnancies). Patterns of association differed using HDD-based case detection, namely PPH and severe PPH cumulative incidence increased over time and there was no correlation between PPH/severe and delivery mode or maternal age. According to HDD, 60.8% (CI; 56.5 to 65.0), 31.5% (CI; 27.6 to 35.7), 7.7% (CI; 5.6 to 10.3) and 12.0% (CI; 9.4 to 15.1) of PPHs were due to uterine atony (O72.1), retained/adherent placenta (O72.0), coagulation defects (O72.3) and late PPH (O72.2) respectively (more than one code per hospitalization being possible).

**Table 2:** Maternal and peripartum characteristics for all 2014–2016 singleton births and for EHR- and HDD-based PPHs and severe PPHs.

	All N (% of column)	EHR-based PPH N (% of row)	HDD-based PPH N (% of row)	EHR-based severe PPH N (% of row)	HDD-based severe PPH N (% of row)
**Total**	7904 (100)	990 (12.5)	533 (6.7)	312 (3.9)	128 (1.6)
**Year**		_**_	_*_		_***_
2014	2529 (32.0)	349 (13.8)	152 (6.0)	99 (3.9)	23 (0.9)
2015	2660 (33.7)	348 (13.1)	167 (6.3)	94 (3.5)	36 (1.4)
2016	2715 (34.3)	293 (10.8)	214 (7.9)	119 (4.4)	69 (2.5)
**Delivery**		_***_			
Vaginal births	5625 (71.2)	797 (14.2)	369 (6.6)	219 (3.9)	91 (1.6)
Cesareans	2279 (28.8)	193 (8.5)	164 (7.2)	93 (4.1)	37 (1.6)
**Maternal age**				_**_	
<35 years	5483 (69.4)	671 (12.2)	355 (6.5)	193 (3.5)	84 (1.5)
≥35 years	2421 (30.6)	319 (13.2)	178 (7.4)	119 (4.9)	44 (1.8)
**Gestational age** ^**Ϯ**^	7875 (99.6)	985 (12.5)	629 (8.0)	310 (3.9)	127 (1.6)
<37 wks.	758 (9.6)	*112 (14.8)	_***_52 (6.9)	**41 (5.4)	_***_20 (2.6)
37–41 wks.	6161 (77.9)	739 (12.0)	380 (6.2)	220 (3.6)	82 (1.3)
>41 wks.	956 (12.1)	134 (14.0)	97 (10.1)	49 (5.1)	25 (2.6)
**Gravidity** ^**Ϯ**^	7887 (99.8)	987 (12.5)	531 (6.7)	310 (3.9)	128 (1.6)
<4	6853 (86.7)	850 (12.4)	*446 (6.5)	*257 (3.8)	112 (1.6)
≥4	1034 (13.1)	137 (13.2)	85 (8.2)	53 (5.1)	16 (1.5)
**Parity** ^**Ϯ**^	7855 (99.4)	983 (12.5)	529 (6.7)	309 (3.9)	128 (1.6)
<3	7580 (95.9)	957 (12.6)	507 (6.7)	295 (3.9)	125 (1.6)
≥3	275 (3.5)	26 (9.5)	22 (8.0)	14 (5.1)	3 (1.1)
**Birthweight** ^**Ϯ**^	7784 (98.5)	982 (12.6)	524 (6.7)	306 (3.9)	124 (1.6)
≤4500g	7741 (97.9)	*972 (12.6)	**516 (6.7)	302 (3.9)	123 (1.6)
>4500 g	43 (0.5)	10 (23.3)	8 (18.6)	4 (9.3)	1 (2.3)
**Transfusion** ≥ 1 PRBCs	149 (1.9)	122 (81.9)	100 (67.1)	105 (70.4)	93 (62.4)
**ΔHb** ^**Ϯ**^	1601 (20.3)	566 (35.4)	393 (24.5)	276 (17.2)	116 (7.2)

EHR: electronic health record; HDD: hospital discharge data; PPH: postpartum hemorrhage; PRBCs: packed red blood cells (one unit = 500 ml, hematocrit 0.6 ± 0.1), ΔHb: peripartum change in hemoglobin

*P≤0.05

** *P* ≤ 0.01

*** *P* ≤ 0.001

using Pearson’s chi-squared test

Ϯ restricted to cases with available variable.

### Case detection accuracy

We reviewed the charts of 183 vaginal deliveries identifying 116 PPHs and 55 severe PPHs and the charts of 149 caesareans identifying 83 PPHs and 54 severe PPHs. Based on chart review and a weighted average of parameters, the estimated cumulative incidence of PPH and severe PPH for the whole population was 12.4% and 3.3%, respectively. For the EHR-based case detection, text mining was able to extract a numeric EBL value in 88% of the 7904 births. In a further 9% of births, EBL was described as normal, and the categorical delivery room EBL was used in the remaining 3%. Accuracy parameters for EHR and HDD case detection compared to reference standard chart review (n = 332) along with those for HDD vs EHR (n = 7904), are described in Table 3. The sensitivity of HDD-based case detection vs reference-standard chart review for PPH was 35.9% (CI; 30.6 to 41.2) in vaginal births and 52.6% (CI; 41.3 to 63.9) in cesareans. Otherwise, sensitivities and specificities were similar in vaginal deliveries and caesareans.

**Table 3:** Accuracy of EHR- and HDD-based case detection vs chart review (n = 332) and of HDD vs EHR (n = 7904), in percent (95% CI).

	Sensitivity*	Specificity*	Kappa
**PPH**			
EHR vs. chart	95.2 (92.6–97.8)	97.1 (92.0–1.0)	-
HDD vs. chart	38.2 (33.3–43.0)	99.4 (98.1–1.0)	-
HDD vs. EHR	42.5 (39.4–45.7)	98.4 (98.1–98.7)	0.51 (0.48–0.54)
**Severe PPH**			
EHR vs. chart	87.5 (85.2–89.8)	99.4 (99.1–99.7)	-
HDD vs. chart	36.2 (26.3–46.1)	99.4 (98.1–1.0)	-
HDD vs. EHR	33.3 (28.1–38.9)	99.7 (99.5–99.8)	0.46 (0.40–0.52)

EHR: electronic health record; HDD: hospital discharge data; PPH: postpartum hemorrhage.

*weighted estimates are presented when comparing to the reference standard to account for stratified sampling.

### Temporal trends

**Fig 1:**
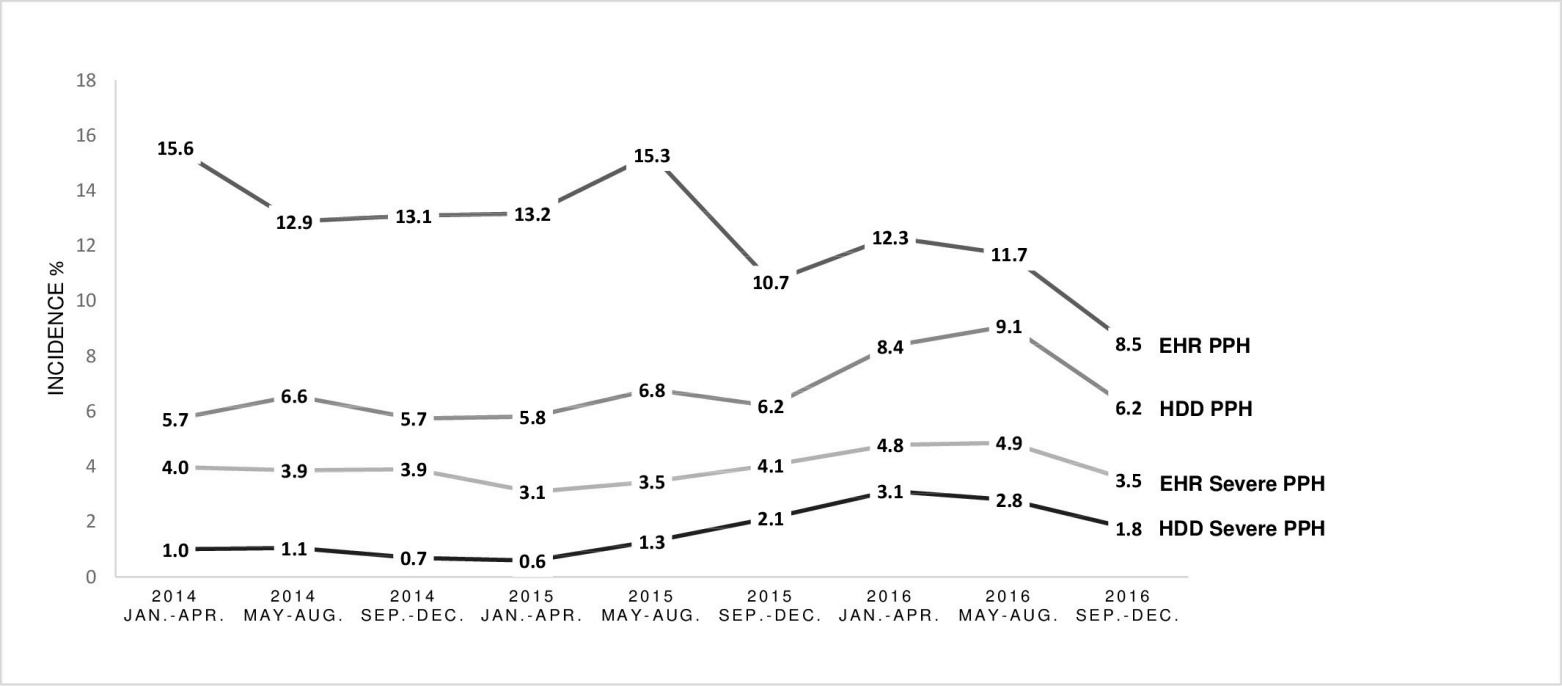
Temporal trends in Postpartum Hemorrhage (PPH) and severe postpartum hemorrhage incidence using Electronic Health Record (EHR) and Hospital Discharge Data (HDD) based case detection, by four-month interval, from 2014 to 2016.

As illustrated in Fig 1, EHR-based PPH cumulative incidence by four-month interval decreased from 15.6% (CI; 13.1 to 18.2) to 8.5% (CI; 6.7 to 10.5) over the three-year period. The cumulative incidence of severe PPH fluctuated around 4% (CI; 3.5 to 4.4). In comparison, HDD underestimated PPH, though a convergence of measures is seen over-time. The adjusted odds of EHR-based PPH, were 6% lower per four-month interval (OR: 0.94 per four-month interval, CI; 0.91 to 0.96, *P* <0.001). The adjusted odds of EHR-based severe PPH did not change significantly over time (OR; 1.01 per four-month interval, CI; 0.97 to 1.06 *P* = 0.574). 149 parturients were excluded from these models due to missing covariate data. HDD-based PPH detection improved over time. In detail, using the EHR-based case detection as a reference standard, the odds of HDD correctly identifying the presence or the absence of PPH increased by 12% per four-month period (OR: 1.12 per four-month interval; CI; 1.09 to 1.16, *P* <0.001). The odds of correct identification of severe PPH did not change significantly over time (OR; 1.04 per four-month interval, CI; 0.99 to 1.09, *P =* 0.134). A sensitivity analysis controlling for maternal age, gravidity, parity, vaginal/caesarean delivery and birthweight produced similar results.

## Discussion

The high sensitivity and specificity of the EHR-based case detection resulted in accurate estimations of PPH/severe PPH cumulative incidence at 13% and 4% respectively. Since changes in Hb can be due to a variety of causes, excluding ΔHb from the definition of non-severe PPH kept false positives low. Furthermore, including ΔHb in combination with a threshold EBL in the definition of severe PPH kept false positive and negative cases to a minimum. On the other hand, HDD performed less well, especially in identifying PPH/severe PPH among those with the condition and substantially underestimated incidence. HDD is not accurate enough and is therefore not recommended for monitoring PPH/severe PPH in this setting.

HDD is still widely used for monitoring and comparing PPH incidence at hospital or country level. However, validation studies for HDD-based PPH indicators remain sparse; existing studies report sensitivities ranging from 28% [26] to 74% [28]. These variations may be due to differences in reference standards (i.e. EBL from EHRs [26] or chart review [28]) as well as local coding accuracy. Concerning PPH incidence, accurate comparisons remain difficult due to differences in definition and data quality [8]. In high income countries alone recent estimates of PPH incidence range from 4% using HDD [20] to 24% using structured EHR data (in a cohort of women undergoing caesareans employing a 1000ml cut-off for PPH [26]).

Our results suggest that improvements in coding practice may in part explain the increase in HDD-based PPH cumulative incidence that has been observed over the last decades in several high-income countries. This increase has not been entirely explained by an increase in risk factors [14–20]. A large retrospective population based study, which used HDD codes to identify PPH, reported an increase in incidence from 2.5 to 4.5% from 1993 to 2014 in Switzerland [20]. Our findings may undermine the validity of these results. Though our study was carried out in only one large university hospital, since coding rules and classifications are the same nationally, and because the sensitivity of HDD-based case detection for PPH and severe PPH was very low (38% and 36%) it can be expected that under-coding is also an issue at other sites in Switzerland. An upcoming Swiss-wide Patient Safety Indicator validation study will help shed further light on this issue. In the meantime and in general, epidemiological conclusions based on HDD should be interpreted with caution in the absence of indicator validation and when consensus in disease definition is lacking.

Concerning limitations of our study, case detection accuracy was determined retrospectively in a relatively limited sample of charts. Though chart review was mainly performed by one physician unclear cases were extensively peer reviewed to obtain consensus regarding the presence or absence of PPH and severe PPH, thus limiting reviewer bias. The limited availability of covariate data did not allow for complex risk-factor adjustment in statistical analyses. Therefore, some of the variation in results over time may be due to case-mix. However, case-mix can be assumed to remain relatively stable within the same hospital with large patient volume, over a three-year period, and probably only had a minimal impact on results. Coding practice, may vary between hospitals, thus limiting the generalizability of our results. Lastly, the procedure codes available for the detection of severe PPH only reflect aggressive treatment; therefore, HDD can be expected to underestimate severe PPH irrespective of coding accuracy.

Our study took place in a university hospital providing secondary and tertiary care. Case-mix along with the objective measurement of blood loss may explain the relatively high prevalence of PPH observed. The observed decrease in the EHR-based PPH cumulative incidence, over the three-year period, may reflect improvements in PPH prevention. The incidence of severe PPH was stable over time and may indicate a need for earlier detection and treatment of PPH to avoid progression towards severity. Adherence to the hospital’s PPH prevention and management guidelines remains unknown. In the future, process indicators may help assess guideline adherence and identify areas for improvement, including the need for evidenced-based strategies to improve maternal safety such as PPH triggers, bundles, protocols and checklists [31]. These tools “(1) are evidence based and can facilitate measurable improvements in quality of care, (2) aid timely diagnosis and treatment to prevent or limit the severity of morbidity, and (3) are customizable for local implementation” [31]. Though coding accuracy for PPH improved over time, HDD majorly underestimated PPH incidence. In Switzerland HDD is required for hospital billing and generated by professional coders who attribute diagnostic and procedure codes solely based on the EHR according to national coding rules and classifications. When coded, PPH due to uterine atony (O72.1), leads to a change in Diagnosis-Related Group and therefore results in higher hospital reimbursement. At the study center, when PPH due to uterine atony is coded, the resulting hospital bill is 1455 CHF higher in vaginal births and 605–905 CHF higher in caesareans compared to cases without PPH or cases with PPH due to other causes. Considering that approximately 77% of PPH cases are due to uterine atony [7] and that only 533 cases were coded compared to 990 detected using the EHR, the hospital lost about 500’000 CHF due to under-coding over this three-year period for the women included in this analysis alone. This represents a major incentive to further improve PPH documentation and coding, especially within the context of cost containment. Under-coding may be related to imprecise or incomplete documentation by physicians in discharge summaries, different terminology used by physicians and coders or the limited ability of coders to interpret physician documentation [32]. We asked an expert physician coder to retrospectively review the coding of 10 EHR-based PPHs and 40 EHR-based severe PPHs undetected by HDD. The majority of under-coding were due to the lack of the explicit mention of PPH in the discharge letter despite EBL being over threshold values or presence of PPH related anemia. Thus, although the information was actually present within the EHR, non-physician coders were unable to accurately translate this information into diagnostic codes, as is illustrated by the high sensitivity of EHR-based case detection when compared to chart review and the low sensitivity of HDD-based case detection when compared to the EHR-based case detection. Consequently quality improvement initiatives have resulted in training a subset of coder; these coders are now specialized in coding for obstetrical issues and are responsible for coding for the obstetrics and neonatal departments. Furthermore, work is underway to automate PPH coding based on the information found in the EHR. Modifying the EHR system by adding a structured field for the presence or absence of PPH or severe PPH that would be filled in by a physician at discharge, could help minimize under-coding be it for manually or automated coding. Further EHR improvements such as numerical documentation of EBL in the delivery room/operation theatre and pictorial blood loss assessment charts [33] to monitor blood loss on the ward may facilitate accurate documentation of cumulative blood loss. Our results indicate that only a minimal dataset, consisting of EBL and changes in peripartum Hb, is necessary to accurately monitor PPH and severe PPH. These data are often available despite differences in EHR systems, presenting a real opportunity to improving the detection, management and coding of PPH, and allowing continuous monitoring of rates and allowing continuous monitoring of rates in a wide range of settings.

## Conclusion

Because of differences in definition and data quality, caution is required when interpreting PPH incidence especially in the absence of indicator validation. Automated algorithms based on structured and textual data from EHRs provide more accurate and timely estimates of PPH incidence than HDD, thus presenting an opportunity in PPH surveillance, facilitating monitoring, comparisons, and therefore improvement of obstetrical safety. They also have the potential to improve hospital reimbursement through better coding in hospital discharge data.

## Supporting information

S1 Fig Inclusion and exclusion flow chart, singleton births 2014–2016 (EBL: Estimated Blood Loss; EHR: Electronic Health Record). (DOCX)

S1 Table Stratified sampling scheme for manual chart review for cesarean births (n = 2297). (DOCX)

S2 Table Stratified sampling scheme for manual chart review for vaginal births (n = 5625). (DOCX)

S3 Table Hospital discharge data codes identifying PPH and severe PPH. (DOCX)

## Abbreviations

EBL: estimated blood loss
EHR: electronic health record
Hb: hemoglobin
ΔHb: changes in hemoglobin
HDD: hospital discharge data
PPH: postpartum hemorrhage
