# Supplementary material for: Hospital discharge data is not accurate enough to monitor the incidence of postpartum hemorrhage
Source: PLoS One. 2021 Feb 3;16(2):e0246119. doi: 10.1371/journal.pone.0246119 (PMC7857548; doi:10.1371/journal.pone.0246119)
Supplement: S1 Fig — (DOCX) [file pone.0246119.s001.docx]

**S1 Fig.** Inclusion and exclusion flow chart, singleton births 2014-2016


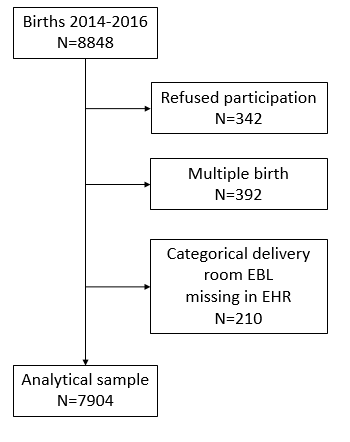


EBL: estimated blood loss; EHR: electronic health record
