# Supplementary material for: Hospital discharge data is not accurate enough to monitor the incidence of postpartum hemorrhage
Source: PLoS One. 2021 Feb 3;16(2):e0246119. doi: 10.1371/journal.pone.0246119 (PMC7857548; doi:10.1371/journal.pone.0246119)
Supplement: S1 Table — (DOCX) [file pone.0246119.s002.docx]

**S1 Table. Manual chart review:** Stratified sampling scheme for cesarean births (n=2297)

| **Estimated blood loss (EBL) in the delivery room (n)** | EBL ≥ 1000 ml  (93) | | | | | | | | EBL < 1000 ml  (2186) | | | | | | | |
| --- | --- | --- | --- | --- | --- | --- | --- | --- | --- | --- | --- | --- | --- | --- | --- | --- |
| **Postpartum drop in Hb (**∆Hb) | ∆Hb >2 g/dl | | | | ∆Hb ≤ 2g/dl or not known | | | | ∆Hb >2 g/dl | | | | ∆Hb ≤ 2g/dl or not known | | | |
| **Factors related to PPH** adherent placenta, manual placenta removal or uterine exploration | Yes | | No | | Yes | | No | | Yes | | No | | Yes | | No | |
| **At least one of the following criteria for severity:**  EBL >1500ml, ∆Hb ≥4 g/dl, transfusions ≥4 PRBCs, embolization, ligature, occlusion, hysterectomy or uterine tamponade (n) | Yes  (8) | No  (7) | Yes  (6) | No  (13) | Yes (11) | No  (6) | Yes (17) | No (25) | Yes  (11) | No (48) | Yes  (28) | No (92) | Yes  (1) | No (626) | Yes  (9) | No (1371) |
| **Sample size for manual chart review (n)** | 8 | 7 | 6 | 13 | 10 | 6 | 10 | 10 | 10 | 10 | 10 | 10 | 1 | 19 | 9 | 11 |
| **Charts positive for PPH (n)** | 8 | 6 | 5 | 13 | 6 | 6 | 9 | 10 | 4 | 3 | 4 | 1 | 1 | 0 | 3 | 0 |
| **Charts positive for severe PPH (n)** | 8 | 2 | 5 | 6 | 1 | 6 | 9 | 5 | 2 | 1 | 4 | 0 | 1 | 0 | 2 | 0 |
| EBL: estimated blood loss; ∆Hb: postpartum drop in hemoglobin; PRBCs: packed red blood cells | | | | | | | | | | | | | | | | |
