# Supplementary material for: Hospital discharge data is not accurate enough to monitor the incidence of postpartum hemorrhage
Source: PLoS One. 2021 Feb 3;16(2):e0246119. doi: 10.1371/journal.pone.0246119 (PMC7857548; doi:10.1371/journal.pone.0246119)
Supplement: S2 Table — (DOCX) [file pone.0246119.s003.docx]

**S2 Table. Manual chart review:** Stratified sampling scheme for vaginal births (n= 5625)

| **Estimated blood loss (EBL) in the delivery room (n)** | EBL ≥ 500 ml  (492) | | | | | | | | EBL < 500 ml  (5133) | | | | | | | |
| --- | --- | --- | --- | --- | --- | --- | --- | --- | --- | --- | --- | --- | --- | --- | --- | --- |
| **Postpartum drop in Hb** **(**∆Hb) | ∆Hb >2 g/dl | | | | ∆Hb ≤ 2g/dl or not known | | | | ∆Hb >2 g/dl | | | | ∆Hb ≤ 2g/dl or not known | | | |
| **Factors related to PPH** adherent placenta, manual placenta removal, uterine exploration or repair of genital injury | Yes | | No | | Yes | | No | | Yes | | No | | Yes | | No | |
| **At least one of the following criteria for severity:** EBL >1000ml, ∆Hb ≥4 g/dl, transfusions ≥4 PRBCs, embolization, ligature, occlusion, hysterectomy or uterine tamponade (n) | Yes  (57) | No (31) | Yes (20) | No (13) | Yes (54) | No (206) | Yes  (18) | No  (93) | Yes  (7) | No (32) | Yes  (6) | No  (18) | Yes  (4) | No (2954) | Yes  (1) | No (2111) |
| **Sample size for manual chart review (n)** | 10 | 10 | 10 | 10 | 12 | 28 | 10 | 10 | 7 | 13 | 6 | 16 | 4 | 16 | 1 | 21 |
| **Charts positive for PPH (n)** | 10 | 10 | 10 | 10 | 12 | 25 | 10 | 8 | 3 | 4 | 4 | 6 | 2 | 2 | 0 | 0 |
| **Charts positive for severe PPH (n)** | 10 | 2 | 10 | 1 | 12 | 2 | 9 | 0 | 3 | 0 | 3 | 1 | 2 | 0 | 0 | 0 |
| EBL: estimated blood loss; ∆Hb: postpartum drop in hemoglobin; PRBCs: packed red blood cells | | | | | | | | | | | | | | | | |
