# Supplementary material for: Hospital discharge data is not accurate enough to monitor the incidence of postpartum hemorrhage
Source: PLoS One. 2021 Feb 3;16(2):e0246119. doi: 10.1371/journal.pone.0246119 (PMC7857548; doi:10.1371/journal.pone.0246119)
Supplement: S3 Table — (DOCX) [file pone.0246119.s004.docx]

| **S3 Table.** Information from hospital discharge data used to detect PPH and severe PPH | | |
| --- | --- | --- |
| **PPH** |  |  |
| ICD-10-GM | Postpartum haemorrhage | O72.0 - O72.3 |
| **Severe PPH (among those with PPH)** | |  |
| CHOP 2014-2016 | Vasopressor infusion | 00.17 |
|  | Transfusion of blood products | 99.00, 99.02-99.04 |
|  | Arterial ligation, occlusion or embolization | 38.86.00, 38.86.10, 38.86.17, 38.86.19, 38.86.99, 39.79.25, 39.79.26, 39.79.35, 39.79.36, 39.79.45, 39.79.46, 39.79.55, 39.79.56, 39.79.6, 39.79.66, 39.79.76, 39.79.86 |
|  | Uterine tamponade | 75.8 |
|  | Hysterectomy | 68.3-68.6 |
| ICD-10-GM | Shock | O751, R571, R579 |
| Discharge | Death | Discharge decision = 5 (death) |
| Transfer | Intensive care after delivery | Number of hours in intensive care > 0 |
| CHOP: Swiss classification of surgical procedures; ICD-10-GM: International Classification of Diseases, 10th Revision, German Modification; PPH: postpartum haemorrhage. | | |
